# Computed Tomographic Pattern of Physiological Intracranial Calcifications in a City in Central Africa

**DOI:** 10.5539/gjhs.v4n1p184

**Published:** 2012-01-01

**Authors:** Uduma Felix Uduma, Fokam Pius, Motah Mathieu

**Affiliations:** Department of Radiology, Abia State University Teaching Hospital Aba, Nigeria Tel: 234-745-0099 E-mail: felixuduma@yahoo.com; Orthopaedic Unit, Department of Surgery, University of Buea Buea, Cameroon Tel: 237-748-77-572 E-mail: piusfokamus@yahoo.com; Neuro-surgical Unit, Department of Surgery, University of Douala Douala, Cameroon. Tel: 237-756-58-643 E-mail:motmath@yahoo.com

**Keywords:** Intracranial calcifications, Pineal, Choroid plexus, Basal ganglia, Computed tomography

## Abstract

**Objective:**

Intracranial calcifications underlie certain brain diseases which may be de novo or systemic. But calclfications un-connected to pathologies are classified physiological.

**Aim:**

To evaluate physiological intracranial calcifications in Douala with establishment of earliest age range of detection.

**Materials and Methods:**

Prospective study of brain computed tomograms was done from April to October 2009 using Schumadzu CT Scan machine. Axial, reconstructed and bone window images as well Hounsfield unit measurements were used for final evaluations. Results were analysed with SSPS 3.

**Results:**

132 patients with 75 males and 57 females were studied and 163 separate calcifications were identified due to co-existent calcifications. The highest calcification was in choroid plexi, constituiting 56.82% of the studied population. This was followed by pineal gland. Both were commonly co-existent with advancing age. These calcifications were first seen at 10-19years. No type of physiological intracranial calcification was seen below age 10. The least calcification of 0.76% of population was in dentate nucleus.

**Conclusion:**

No intra-cranial physiological calcifications started earlier than 9years in Douala, a city in Cameroon, Central Africa.

## 1. Introduction

Intracranial physiological calcifications are unaccompanied by any evidence of disease and have no demonstrable pathological cause (Daghighi *et al*, 2007, pp115-9). They are due to calcium and sometimes iron depositions in the blood vessels of the different structures of the brain (Daghighi *et al*, 2007, pp115-9). CT remains the imaging modality of choice in the detection of two processes: acute intracranial haemorrhage and calcifications (Glo & Zee, 1998, pp542-58). Computed tomography is a veritable imaging modality for best identification and characterizations of any intracranial calcifications (Sarmiento de La Iglesia *et al*, 2006, pp19-26, Rozylo-Kalinowaska *et al*, 2003, pp602-5). It is also the most sensitive means of detections of these calcifications (Daghighi *et al*, 2007, pp115-9). A number of factors including slice thickness, window width, and level may affect the detectability of calcification on CT (Glo & Zee, 1998, pp542-58). It is superior to conventional radiography in this respect as the specific regional localisation of the calcification can be ascribed. Besides, most calcifications are not visualized on plain radiographs especially if the CT attenuation values are less than 200 Hounsfield units (HU) (Patel, 1987, pp177-80). CT is superior to MRI in the detection of calcifications as. MRI cannot reliably rule out or determine the presence of calcifications (Glo & Zee, 1998, pp542-58 (Sarmiento de La Iglesia *et al*, 2006, pp19-26). Also, the magnetic resonance imaging findings for intracranial calcifications previously demonstrated at CT are variable and unspecific (Glo & Zee, 1998, pp542-58 (Sarmiento de La Iglesia *et al*, 2006, pp19-26). The most frequent appearance of intracranial calcifications on T1W sequence is an area isointense with the cerebral cortex while most frequent appearance on T2W sequence is focus of hypointensit (Sarmiento de La Iglesia *et al*, 2006, pp19-26).

Depositions of calcium salts in intracranial structures forms calcifications (Rozylo-Kalinowaska, *et al.*, 2003, pp602-5). Intra-cranial calcifications can be sub-divided into physiological or pathological (Chapman& Nakiel, 2003, p428). Physiological intracranial calcifications are seen in sites like pineal gland, choroid plexus, basal ganglia, lens, cerebellum, ligaments, dura, vessels, arachnoid granulations and habenula.(Daghighi *et al.*, 2007, pp115-9, Dahnert,2003, p237).

Physiological intracranial calcifications are asymptomatic and detected by neuro-imaging (Basak, 2009, pp 220-224). They may have no clinical importance but may be critical findings in diagnosing underlying pathology (The Intracranial calcifications, The Free-online Librarary),. Most importantly, these statistics can be used in comparing physiological and pathological intracranical calcifications (Daghighi *et al*, 2007, pp115-9). To the best of our knowledge, the publications on this topic is scant in this African region, encouraging us in pursuit of this study.

To evaluate the pattern of physiological intracranial calcifications in Douala and observation of earliest age range of each calcification

## 2. Materials and Methods

A prospective study of cranio-cerebral CT done from 8/4/09 to 18/10/2009 was undertaken in Department of Radiology, Polyclinic Bonanjo, Douala, Cameroon, (a tertiary health care provider). This was done using Schumadzu CT scan machine with continuous rotational system. Axial sections were done using slice tissue thickness of 2mm from the base of the skull to the sella turcica, thence 5mm from the sella to the vertex. IV Iopamidol at 1mm/kg was given when indicated. Images were reconstructed to achieve sagital and coronal images. Hounsfield unit (HU) measurement and bone window were employed in some cases of doubt so as to differentiate calcifications from acute haemorrhage. The HU of calcifications is above 100HU while HU of acute haemorrhage is in the range of 60-90HU taking into account the effect of partial volume averaging. The structures evaluated consisted of (a) the pineal gland, (b) the choroid plexus, (c) the habenula, (d) the basal ganglia, (e) the tentorium cerebelli, sagittal sinus and falx cerebri, (f) vessels and (g) lens and other structures which could be calcified. Patients consent was obtained. The ethical board review committee’s approval was obtained. All patients with any pathology linked or associated with intra-cranial calcifications and those with improper data documentation were excluded. Results were analysed using SSPS 3.

## 3. Results

132 of 174 brain computed tomograms were considered optimal for our study and analysed. There were 75 males (56.82%) and 57 females (43.18%) with age range of 0.4-81years and mean age of 45.5years. Largest population studied was 38 in 40-49 year age range with 24 males and 14 females. This age range also had the highest number of intra-cranial calcifications of 58 (35.58%) of the total number of 163 intracranial calcifications detected in this study. In this 40-49years, 40 (68.96%) calcifications were seen in males and 18 (31.04%) females with male: female ratio 2.22:1. This is followed by 26 calcifications (14 in males, 12 in females with ratio of male to female 1.6:1) in the 50-59 age range. 8 calcifications (2 in males and 6 in females with male to female ratio 1:3) were seen in 80-89 year age range. No calcification was seen below 10 years of age in both sexes. In older age of 70 years and above, females had more calcifications whereas from 69years and below, males had more calcifications. The highest number of calcifications of 75, constituting 46.01% of total number of detected calcifications and 56.82% of total studied populations was seen in the choroid plexus (atria), with 44 calcifications in males and 31 in females (male: female ratio is 1.42:1). Highest number of choroid calcification 24 (32%) was in the 40-49 year age range with male predominance followed by 15 in the 50-59years with female predominance.

Pineal gland calcifications were 61 (37.42%) of all calcifications and 46.21% of studied population. It was the second highest with 36 cases in males and 25 in females (male: female 1.44:1). Peak age is also 40-49years followed by 50-59 years. Least calcifications of 1 case were in the dentate nucleus in a female. The earliest age of calcification of choroid plexus/pineal gland in males was in 10-19 year age range whereas earliest calcifications of choroid plexus/pineal gland in females were 20-29 years. Both show equal predominance of choroid plexus/pineal calcifications with increasing age, females tend to have increasing pineal gland calcifications than males.

Anterior falx calcifications (21) is more than posterior falx calcification 3 (anterior to posterior 7:1). Both show male predominance (anterior falx male: female ratio 1.6:1) and (posterior falx male: female ratio 2:1). Earliest calcification of anterior falx started at 20-29 year age range. Highest incidence is also at 40-49 years, with 6 cases in males and 3 cases in females (male to female ratio 2:1). Posterior falx calcifications started at 40-49 years.

## 4. Discussion

Intra-cranial calcifications can be physiological or pathological. When physiological, it is asymptomatic and detected incidentally in neuro-imaging (Verulashirili *et al*, 2006, pp 39-43). Intracranial calcifications seen on computed tomography (CT) are the most common finding in the everyday practice of neuroradiology, because noncontrast-enhanced CT of the head is the preferred imaging modality worldwide for the initial evaluation of patients with acute or chronic neurological problems (The Intracranial calcifications, The Free-online Librarary). Possible sites of physiological calcifications are pineal gland, choroid plexi, habenula, dura (falx cerebri, tentorium, vault), ligaments (petro-clinoid, interclinoid), dura, pacchonian bodies, basal ganglia, cerebellum, pituitary gland, and lens (The Intracranial calcifications, The Free-online Librarary, Sutton, 1995, p746, Medical definitions-online). This physiological calcifications is thought to be an adaptive metabolic processes which depend on many factors, among which include the individual constitutional ground and aging (Guja *et al*, 2005, pp1-8).

In this study, understandably no intracranial calcifications of any type was seen in the young age range of 0-9 years as in other studies (Guja *et al*, 2005, pp1-8). Presence of pineal calcifications in a child less than 6 years suggests neoplasm (Dahnert, 2003, p237, Medical definition, online). Doyle and Anderson, 2006, pp822-6) observed 1% of pineal calcifications in those less than 6years (Menon & Harinarayan, 2009, pp55-60). Males started choroid plexus calcification earlier than females in this study, 10-19years and 20-29years respectively. Evidence of choroid plexus calcifications has been recorded in 9.5% of children from 9 to 15 years of age in some reports. (The Intracranial calcifications, The Free-online Librarary).

Individual calcifications were 163 from 132 patients. This is because a single patient can have multiple calcifications. Such co-existence was commonly between pineal gland and choroid plexus and between anterior and posterior falx cerebri. Choroid plexus calcification is known to be associated with pineal gland calcification Doyle and Anderson, 2006, pp822-6)

In this study, the commonest calcifications noted were choroid plexi and pineal glands 46.01% (56.82% of total population) and 37.42% (46.21% of total population) respectively. This choroidal plexus calcification predominance has been reported by other authors (Menon & Harinarayan, 2009, pp134-135). However a reversal of this pattern was noted by other studies (Daghighi *et al*, 2007, pp115-9; Admassie & Mekonne, 2009, pp55-60). Admassie & Mekonne, 2009, pp55-60Admassie and Mekonne reported an overall incidence of normal pineal gland calcifications of 72.0% and that of choroid plexus 43.3%. Similarly, Daghighi *et al* observed 71% of their studied population had pineal gland calcifications while 66.2% had choroid plexus calcifications (Daghighi *et al*, 2007, pp115-9). Both choroid and pineal calcifications in this study peaked at 40-49 years with male predominance. The physiologic calcifications of the choroid plexus are very common after the age of 40 years (The Intracranial calcifications, The Free-online Librarary).

The pattern of choroid plexi calcification in this study was symmetrical and bilateral in 100% of positive cases of intracranial calcifications. Such calcification increased with age with maximum of 80% in 80-89 years. Choroid plexi calcifications are known to occur in all ventricles, most commonly in the glomus within the atrium of lateral ventricles near foramen of Monro. Other sites are tela choroidea of third ventricles, roof of fourth ventricle along foramen of Luschaka (Dahnert, 2003, p237). In this study, all the calcifications were in the atria of lateral ventricles.

Physiologic pineal calcification is more common in children than previously reported, mostly because of improving computed tomography technology. The pathogenesis of pineal gland calcifications is that the pineal organ (pineal gland, epiphysis cerebri) contains several calcified concretions called “brain sand” or acervuli corpora arenacea. (Vigh *et al*, 1998, pp851-70) Predominantly composed of calcium and magnesium salts, corpora arenacea are numerous in old patients. In smaller number they can be present in children as well. (Vigh *et al*, 1998, pp851-70). Corpora arenacea occur not only in the actual pineal tissue but also in the leptomeninges, habenular commissure and in the choroid plexus (Vigh *et al*, 1998, pp851-70). The size of physiological pineal calcification is usually 3-5 mm, if greater than 1 cm, raise concerns for underlying tumor, like pinealoma, teratoma, AV malformation (Medical definition, online). Usually, pineal gland calcifications are in the form of cluster of amorphous, irregular densities or it may be solitary (Medical definition, online). But the incidence of pineal calcification noted in this study across all ages was 46.21% of the population compared to 2/3_rd_ of the population in some literature (Dahnert, 2003, p237; Admassie & Mekonne, 2009, pp55-60). Some literatures recorded 40% of pineal calcification at 20 years, but only 15.79% of our studied population below 20years and 30% below 30years had physiological pineal gland calcifications (Dahnert, 2003, p237, The Free-online Library). The pattern of pineal calcification across ages in this study is that females showed more calcifications in older age group of 70 years and above whereas males had more calcifications below 69 years. The plausible explanation is the complete removal of the effect of the female sex hormonal control. The incidence of pineal gland and choroid plexus calcifications show male bias in this study as in other studies. The incidence of normal pineal gland and choroid plexus calclfications were higher in males than females by 13.1% and 6.0% respectively Admassie & Mekonne, 2009, pp55-60). The frequency of pineal gland and choroid plexus calcifications show a steady increase in both sex groups Admassie & Mekonne, 2009, pp55-60).

The only dura calcifications in this study were in the falx cerebri. The anterior to posterior falx calcification ratio is 7:1. Males show predominance in the above mentioned two dural calcifications. 15.91% of this studied population had falx calcification compared to 10% of populations in some studies (Dahnert, 2003, p237. Anterior falx calcification was first noted at above 19years (compare to >3years in some literatures) while posterior falx started at a later age of 40-49 (Dahnert, 2003, p237). Displacement of falx calcifications has been a good indicator of raised intra-cranial pressure or intracranial mass lesion in adults in earlier days of only conventional radiography. This is because physiologic calcifications of the dura are very common in older age groups and are usually located in the falx or the tentorium (The intracranial calcifications, The Free-onlineLibrary).. Presence of dural calcifications in children should raise the suspicion of underlying pathology, mainly basal-cell nevus syndrome. (The intracranial calcifications, The Free-onlineLibrary)

Brain calcinosis syndrome (BCS) is usually defined as bilateral calcium accumulation in the brain parenchyma, most often within the basal ganglia. Various terms have been used to describe basal ganglia calcification including calcification(s) of the basal ganglia, basal ganglia calcification(s), Fahr syndrome, intracranial calcification, pallidal calcification, and striopallidodentate calcinosis (Basak, 2009, pp220-224). More than 50 reported clinical conditions have been associated with BCS, including sporadic entities and the heredofamilial conditions (Basak, 2009, pp220-224). Basal ganglia are supplied by perforating arteries which are prone to small vessel ischaemia with increasing age (Rossi *et al*, 1993, 192-8). The review of literature shows, now, that there is no definite pathogenesis of basal ganglia calcifications (Rossi *et al*, 1993, 192-8). The calcifications in the basal ganglia are usually punctate and are located within the globus pallidus, the head of the caudate nucleus, and the putamen and are very common in middle-aged individuals and the elderly. The intracranial calcifications, The Free-online Library), Computed tomography is superior to conventional skull radiographs in detecting basal ganglia (Rossi *et al*, 1993, 192-8). Basal ganglia calcifications have been associated with different conditions, abnormality with calcium-phosphorus metabolism being the most of these associations (Rossi *et al*, 1993, 192-8). Basal ganglia calcifications incidence of 1.52% in our studied population is within the known range of 0.3-1.5% (Basak, 2009, pp220-224, Verulashvili *et al*, 2006, pp39-43). Though lower values of 0.8% in other studies has been recorded (Daghighi *et al*, 2007, pp115-9). The earliest basal ganglia calcification was first noted at middle age of life (40-49years) in this study which agrees with other assertions (The intracranial calcifications, The Free-online Library). The presence of basal-ganglia calcifications in patients <30 years of age should prompt careful clinical evaluation to rule out another etiology like hyperparathyroidism, hypoparathyroidism, congenital disorders such as Fahr syndromp (Intracranial calcifications, The Free-online Library). In general, pathological basal ganglia calcification is due to various causes such as congenital, metabolic disorders, idiopathic, aging neuro-degenerative(Fahr syndrome, Cockayne syndrome), infectious (cytomegalovirus, toxoplasmosis) and genetic disorders, birth anoxia, radiation, lead and carbon monoxide poisoning and others (Basak, 2009, pp220-224; The Free-online Library; Menon & Harinarayam, 2009, pp134-135; Rossi *et al*, 1993, 192-8; Erdem *et al*, 1994 pp111-22). Hypoparathyroidism and pseudohypoparathyroidism are the most common causes of pathological basal ganglia calcification (Basak, 2009, pp220-224; Verulashvili *et al*, 2006, pp39-43). Before CT, 70% to 80% of brain calcification detected on plain skull radiography was associated with hypoparathyroidism (Medical definition, online).

All types of intracranial calcifications increased at old age except for lens and other non-defined calcifications (Daghighi *et al*, 2007, pp115-9). The calcification of the intra-cavernous segment of internal carotid artery (ICA) or para-sellar ICA is thought to be due to intima carotid siphon (double –bent shape of the para-sellar ICA) (Weninger *et al*, 1999, pp85-97). Physiologic calcifications can be seen in the cerebellum, with the dentate nucleus being the most common site as noted in this study (The intracranial calcifications, The Free-online Library).

## 5. Conclusion

No any type of intracranial calcification was seen below 10 years of age in Douala. The commonest physiological intracranial calcification is choroid plexi, followed by pineal gland. Both calcifications started at 10-19 year age range and both also show male predominance, Choroid plexus calcifications were all bilateral and symmetrical. Choroid plexus and pineal gland calcifications were co-existent with advancing age. The least calcification is in the dentate nucleus of cerebellum.

**Table 1:** Showing the numbers of intracranial calcifications at different locations in age groups and sex

Age range	Ant	falx	Post	falx	Choroid	plex	Pineal	gland	Basal	gland	Dentate	nucl	Habenu	Liga	Arach	Lens	Total
	M	F	M	F	M	F	M	F	M	F	M	F				F	
0-9	-	-	-	-	-	-	-	-	-	-	-	-	-	-	-	-	-
10-19	-	-	-	-	3	-	3	-	-	-	-	-	-	-	-	-	6
20-29	2	1	-	-	6	1	5	1	-	-	-	-	-	-	-	-	16
30-39	1	2	-	-	5	4	3	4	-	-	-	-	-	-	-	-	19
40-49	6	3	1	-	16	8	16	7	1	-	-	-	-	-	-	-	58
50-59	2	-	-	-	7	8	5	4	-	-	-	-	-	-	-	-	26
60-69	1	-	-	-	3	2	1	2	-	-	-	-	-	-	-	-	9
70-79	1	1	1	1	3	5	2	4	-	1	1	-	-	-	-	-	21
80-89				-	1	3	1	3	-	-	-	-	-	-	-	-	8
Total	13	8	2	1	44	31	36	35	1	1	1	-	-	-	-	-	163
Distribution of Studied Population																	
Age Ran					Males				Females					Total			
0-9					8				2					10			
10,-19					5				4					9			
20-29					8				3					11			
30-39					4				13					17			
40-49					24				14					38			
50-59					14				8					22			
60-69					6				2					8			
70-79					4				8					12			
80-89					2				3					5			
90-99					0				0					0			
Total					75				57					132			

**Table 2:** Showing total number calcifications pattern across age ranges

Age Range	Males	Females	
0-9	0	0	0
10--19	6	0	6
20-29	13	3	16
30-39	9	10	19
40-49	40	18	58
50-59	14	12	26
60-69	5	4	9
70-79	7	14	21
80-89	2	6	8
90-99	0	0	0
Total	96	67	163

**Figure 1:**
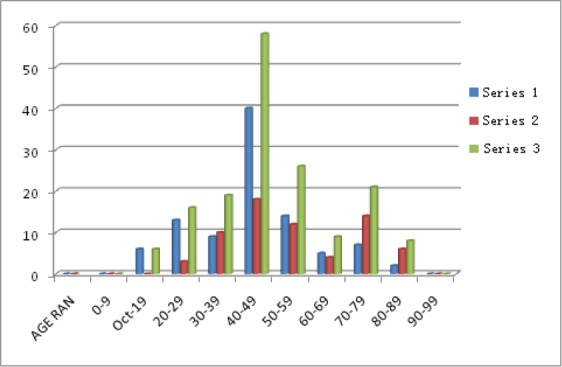
Calcification distributions across age ranges Series 1=Males, Series 2=Females, Series 3=Total

**Figure 2:**
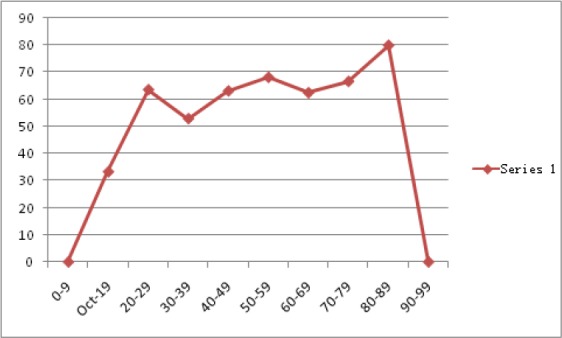
Choroid plexus graphical representations of %calcification pattern across age ranges

**Figure 3:**
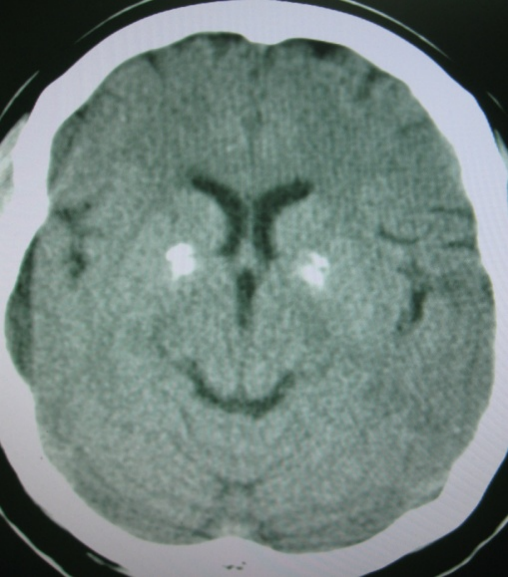
Axial brain ct showing bilateral symmetrical basal ganglial calcification

**Figure 4:**
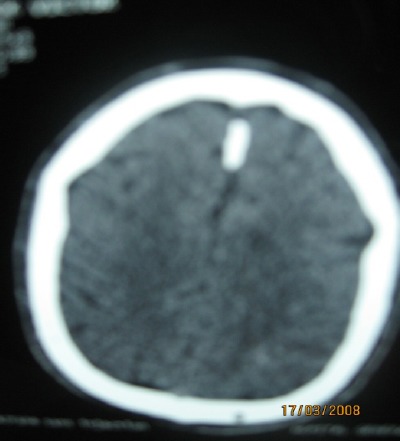
Axial brain ct showing anterior falx calification

**Figure 5:**
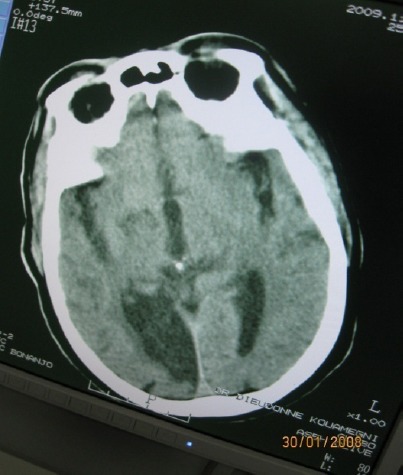
Axial brain ct showing pineal gland calcification

**Figure 6:**
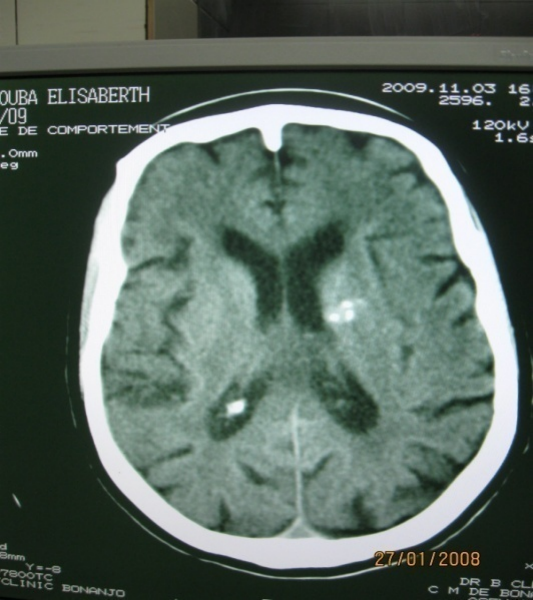
Axial brain ct showing choroid plexus and left basal ganglial calcifications
